# Cloning, Expression, and Characterization of a Psychrophilic Glucose 6-Phosphate Dehydrogenase from *Sphingomonas* sp. PAMC 26621

**DOI:** 10.3390/ijms20061362

**Published:** 2019-03-18

**Authors:** Kiet TranNgoc, Nhung Pham, ChangWoo Lee, Sei-Heon Jang

**Affiliations:** Department of Biomedical Science and Center for Bio-Nanomaterials, Daegu University, Gyeongsan 38453, Korea; trankiet.hust@gmail.com (K.T.); ptnhung.hust@gmail.com (N.P.); leec@daegu.ac.kr (C.L.)

**Keywords:** glucose 6-phosphate dehydrogenase, cold adaptation, psychrophilic enzyme, NADP^+^-preferring enzyme, Arctic, kinetics, conformational flexibility

## Abstract

Glucose 6-phosphate dehydrogenase (G6PD) (EC 1.1.1.363) is a crucial regulatory enzyme in the oxidative pentose phosphate pathway that provides reductive potential in the form of NADPH, as well as carbon skeletons for the synthesis of macromolecules. In this study, we report the cloning, expression, and characterization of G6PD (SpG6PD1) from a lichen-associated psychrophilic bacterium *Sphingomonas* sp. PAMC 26621. SpG6PD1 was expressed in *Escherichia coli* as a soluble protein, having optimum activity at pH 7.5–8.5 and 30 °C for NADP^+^ and 20 °C for NAD^+^. SpG6PD1 utilized both NADP^+^ and NAD^+^, with the preferential utilization of NADP^+^. A high *K*_m_ value for glucose 6-phosphate and low activation enthalpy (Δ*H*^‡^) compared with the values of mesophilic counterparts indicate the psychrophilic nature of SpG6PD1. Despite the secondary structure of SpG6PD1 being maintained between 4–40 °C, its activity and tertiary structure were better preserved between 4–20 °C. The results of this study indicate that the SpG6PD1 that has a flexible structure is most suited to a psychrophilic bacterium that is adapted to a permanently cold habitat.

## 1. Introduction

Glucose 6-phosphate dehydrogenase (G6PD) is a ubiquitous enzyme from prokaryotes to eukaryotes that catalyzes the first reaction in the pentose phosphate pathway (PPP) wherein glucose 6-phosphate (G6P) is converted to 6-phosphoglucono-δ-lactone while reducing NAD(P)^+^ to NAD(P)H. NADPH provides reducing power for the synthesis of lipid and aromatic amino acids [1], and also serves as a cofactor of several oxidoreductases, including glutathione reductase and thioredoxin reductase, which protect cells from oxidative damage [2,3].

While numerous G6PDs from thermophiles and mesophiles are well characterized [4,5,6,7,8], only a small number of G6PDs from psychrophiles are known [9,10,11]. Antonietta et al. showed that although G6PDs from cold-adapted fish have a molecular similarity with their mesophilic counterparts, their biochemical characteristics were very different from those of mesophiles, which made the enzymes more appropriate to cold-adapted organisms [11]. The activity of G6PD from *Chlorella ellipsoidea* markedly increased under freezing conditions, and also stimulated the freezing tolerance of *Chlorella vulgaris* [12,13]. Interestingly, the enhanced viability of *Saccharomyces cerevisiae* harboring the G6PD gene from *Chlorella vulgaris* C-27 (CvG6PD) was observed during the freeze-thaw process as compared with control yeast cells lacking the CvG6PD gene [12]. Conversely, the catalytic performance of G6PD from the blood cells of Antarctic fish was higher than those of humans, which is likely to be a factor for cold adaptation of the fish in Antarctica [10]. These results indicate that G6PD is not only important for glucose metabolism and NADPH/NADH generation but also plays a crucial role in the cold adaption of organisms in extremely low-temperature habitats.

To gain insight into the cold adaptation mechanism of psychrophilic G6PDs, we cloned, expressed, and characterized a psychrophilic G6PD (SpG6PD1) from the *Sphingomonas* sp. PAMC 26621, which was isolated from an Arctic lichen *Cetraria* sp. on Svalbard islands [14]. 

## 2. Results

### 2.1. Gene Cloning of SpG6PD1

**Figure 1:**
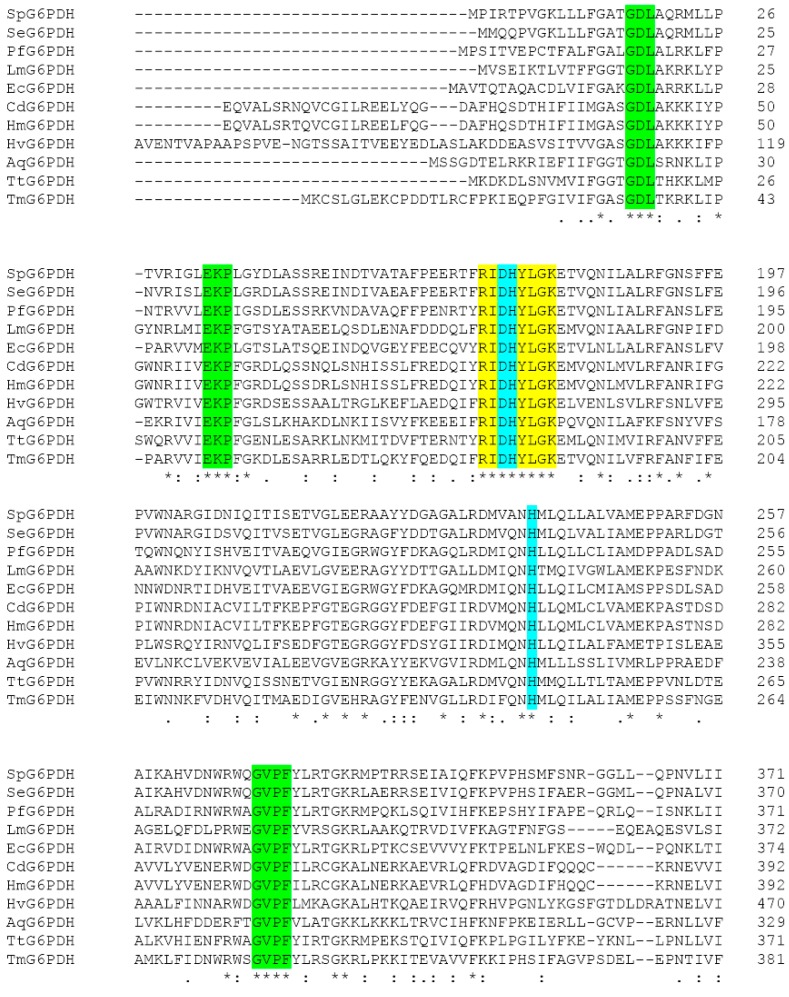
Multiple alignments of glucose 6-phosphate dehydrogenase (G6PD) sequences. SpG6PD1 (*Sphingomonas* sp. PAMC 26621), SeG6PD (*Sphingomonas elodea* [20]), PfG6PD (*Pseudomonas fluorescens* [21]), LmG6PD (*Leuconostoc mesenteroides* [5]), HmG6PD (Human [22]), EcG6PD (*Escherichia coli* [23]), CdG6PD (*Camelus dromedarius* [24]), HvG6PD (*Hordeum vulgare* [25]), AqG6PD (*Aquifex aeolicus* VF5 [7]), TtG6PD (*Thermoanaerobacter tengcongensis* [6]), TmG6PD (*Thermotoga maritima* [26]). Catalytic residues Asp174, His175, and His237 (turquoise color), the substrate-binding motifs (yellow color), cofactor-binding pockets (green color).

The *zwf* gene with a 1467-bp open reading frame was cloned from the genome of *Sphingomonas* sp. PAMC 26621. The SpG6PD1 has a calculated molecular weight of 55.6 kDa and an isoelectric point of 5.71. The amino acid sequence alignment of SpG6PD1 with those of 10 other G6PDs showed the highest identity with *Sphingomonas elodea* (88% identity) followed by *Pseudomonas fluorescens* (49% identity) and *Escherichia coli* (46% identity) (Figure 1 and Supplementary Table S1).

Moreover, the catalytic triad (Asp174, His175, and His237), the substrate-binding site (RIDHYLGK), and the cofactor-binding pockets were conserved in SpG6PD1 as reported in *L. mesenteroides* [15,16,17,18,19] (Figure 1). The structural model of SpG6PD1 was constructed using the crystal structure of *L. mesenteroides* G6PD as a template using the Swiss model, which predicted that SpG6PD1 was a homodimer enzyme.

### 2.2. Expression and Purification of SpG6PD1

**Figure 2:**
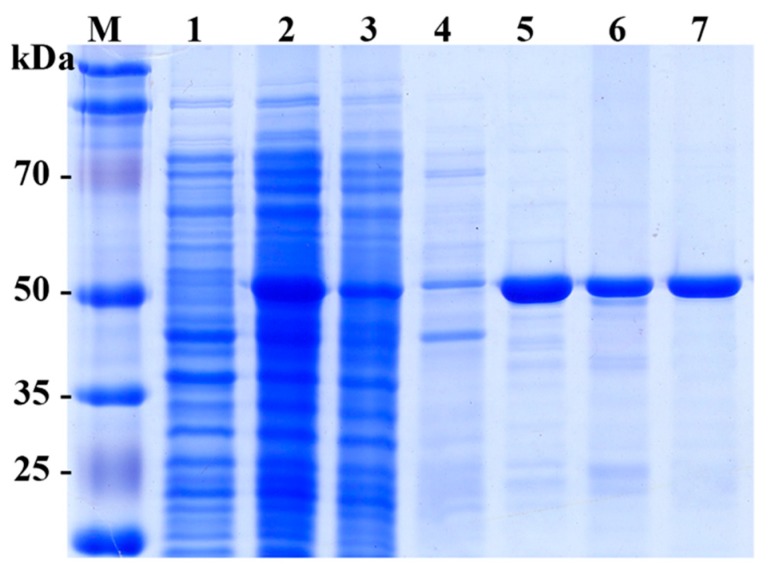
SDS-PAGE analysis of expression and purification of SpG6PD1. M: molecular weight marker, lane 1: control (uninduced cell), lane 2: cell lysate, lane 3: flow-through, lane 4: wash, lane 5–6: eluted fractions from HisTrap column, lane 7: eluted fraction from Q-Sepharose column.

The recombinant SpG6PD1 with a C-terminal six His-tag was expressed in *E. coli* BL21(DE3) as a soluble protein (Figure 2). SpG6PD1 was purified to homogeneity with an overall yield of 57% and an 8.7 purification fold compared to the cell lysate (Table 1). 

The recombinant SpG6PD1 protein appeared as a 56-kDa protein on an SDS-PAGE gel (Figure 2).

### 2.3. Optimum pH and Temperature

**Figure 3:**
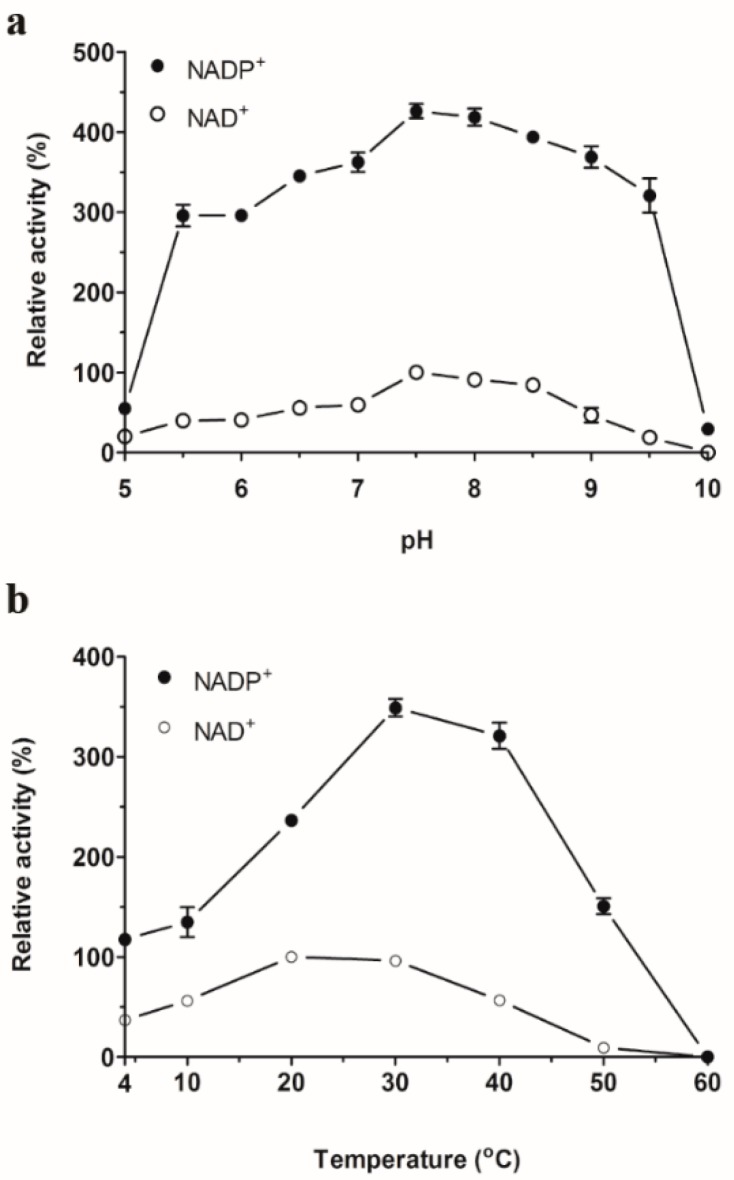
Apparent optimum pH and temperature of SpG6PD1. (**a**) Effect of pH: SpG6PD1 activity was measured at a range of pH between 5.0–10.0, for 2 min at 30 °C. 100% = 27.6 µM·min^−1^. (**b**) Effect of temperature: SpG6PD1 activity was evaluated in the reaction mixture at various temperatures, from 4–60 °C for 2 min. 100% = 32.1 µM·min^−1^. Data correspond to the value ± SD of three independent experiments.

The optimal pH of SpG6PD1 was determined to be between pH 7.0–8.5 in both NADP^+^-linked and NAD^+^-linked reactions, with the highest activity observed at pH 7.5 (Figure 3a). This optimal pH range of SpG6PD1 is similar to other G6PDs [6,10,27]. SpG6PD1 displayed optimal temperatures at 30 °C for NADP^+^-linked reactions and 20 °C for NAD^+^-linked reactions (Figure 3b); these temperatures are higher than the physiological growth temperature of 15 °C for *Sphingomonas* sp. PAMC 26621, which is a feature often found in cold-adapted enzymes [28].

### 2.4. Effect of Metal Ions on the SpG6PD1 Activity

SpG6PD1 activity was slightly enhanced by adding divalent cations Mg^2+^ and Ca^2+^. However, the activity was inhibited by Zn^2+^, Fe^3+^, Cu^2+^, and Ni^2+^ at concentrations of both 1 mM and 5 mM (Table 2); to our surprise, the activity completely disappeared at 5 mM Ni^2+^. The addition of Mn^2+^ or ethylenediaminetetraacetic acid (EDTA) had no effect on SpG6PD1 activity. These results illustrate that metal ions are not required for the activity of SpG6PD1. Effects of metal ions on G6PD activity have been reported previously in numerous studies [6,7,29,30,31].

### 2.5. Thermal Stability

**Figure 4:**
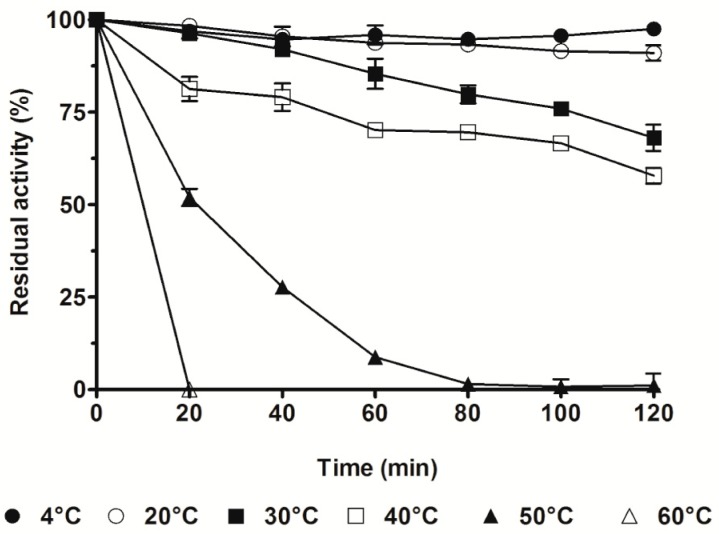
Thermal stability of SpG6PD1. SpG6PD1 enzyme in buffer A was aliquoted and then incubated at 4, 20, 30, 40, 50, and 60 °C for the indicated time, after which SpG6PD1 activity was measured in the reaction mixture with NADP^+^ as a cofactor, at 30 °C for 2 min. The catalytic activity at 30 °C before incubation was considered 100%. Data correspond to the value ± SD of three independent experiments.

The thermal stability of SpG6PD1 was determined by measuring the enzymatic activity at the optimal temperature (30 °C) with NADP^+^ as a cofactor, and incubation at various temperatures (4–60 °C). We observed that the activity of SpG6PD1 maintained between 4–20 °C for 2 h. However, at 30 °C and 40 °C, the activity decreased to 68% and 58% of the initial activity, respectively. Interestingly, when exposed for 20 min, the SpG6PD1 activity rapidly reduced at 50 °C and completely lost at 60 °C (Figure 4).

### 2.6. Kinetics and Thermodynamic Analysis

The determined kinetic and thermodynamic parameters of SpG6PD1 are presented in Table 3. 

In both NADP^+^-linked and NAD^+^-linked reactions, the *K*_m_ and *k*_cat_ values at 30 °C are higher than those at 20 °C. In NADP^+^-linked reactions, SpG6PD1 shows a *K*_m_ value of 145 µM for G6P (*K*_m_G6P) and a *k*_cat_ value of 8081 min^−1^ (Table 3). In contrast, in the NAD^+^-linked reaction, the *K*_m_G6P value increases to 675 µM (4.7-fold higher than the *K*_m_G6P value of the NADP^+^-linked reaction), whereas the *k*_cat_ value (1196 min^−1^) is 6.8-fold lower than that obtained with the NADP^+^ reaction (Table 3). As a consequence, the catalytic efficiency (*k*_cat_/*K*_m_) of the NADP^+^-linked reaction is 31-fold higher than that of the NAD^+^-linked reaction (Table 3). The thermodynamic parameters reveal that the Δ*G*^‡^, Δ*H*^‡^, and Δ*S*^‡^ values in the NADP^+^-linked reaction are lower than the NAD^+^-linked reaction (Table 3). Particularly, the activation energy (*E*a) for the NADP^+^ reaction (30.3 kJ·mol^−1^) is less than twice the *E*a for the NAD^+^-linked reaction (56.5 kJ·mol^−1^). Conversely, the Δ*H*^‡^ and Δ*S*^‡^ values obtained for SpG6PD1 are lower than its cold-adapted, mesophilic, and thermophilic counterparts (Table 4).

### 2.7. Spectroscopy Analysis

**Figure 5:**
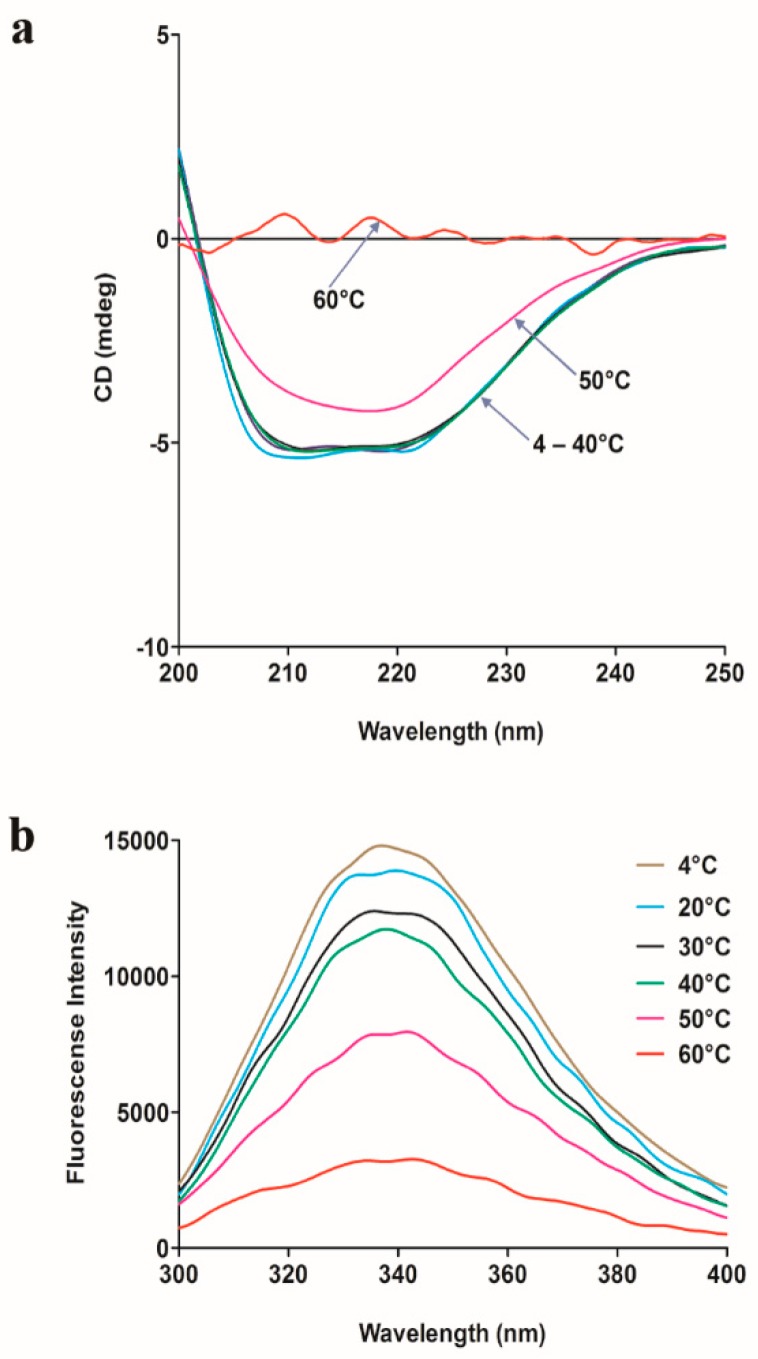
Spectroscopic analysis of SpG6PD1. (**a**) Circular dichroism (CD) spectra of SpG6PD1: The CD spectra were measured at 25 °C after incubating a 0.3 mg/mL protein sample at 4, 20, 30, 40, 50, and 60 °C for 1 h. (**b**) Fluorescence spectra of SpG6PD1: the fluorescence spectra were measured at 25 °C after incubating 1 μM of the enzyme at 4, 20, 30, 40, 50, and 60 °C for 1 h upon excitation at 280 nm.

The effect of temperature on the secondary structure and tertiary structure of SpG6PD1 was evaluated by measuring the circular dichroism (CD) spectra in the far-UV region (200–250 nm) and the intrinsic protein fluorescence upon excitation at 280 nm, respectively. The spectroscopic results show that SpG6PD1 maintains the tertiary structure at 4–20 °C, whereas the secondary structure is maintained between 4–40 °C; both structures are completely denatured at 60 °C after 1 h of incubation (Figure 5).

This data suggests that SpG6PD1 is a thermolabile enzyme. Furthermore, the CD spectra show maximum absorption peaks at 222 and 208 nm, indicating that SpG6PD1 is a protein containing an abundant α-helix [32].

## 3. Discussion

Numerous G6PDs isolated from mesophiles and thermophiles have been characterized [4,5,6,7,8,22,24,25,27,30,33], but only a few psychrophilic G6PD are known [9,10]. As a result, the functioning mechanism of psychrophilic G6PDs at low temperatures remains largely unraveled. In the present study, we show that the psychrophilic SpG6PD1 from Arctic bacterium *Sphingomonas* sp. PAMC 26621 maintains activity and structure between 4–20 °C as a psychrophilic enzyme. The kinetic and thermodynamic data reveal high *K*_m_ values and low activation enthalpy compared to its mesophilic and thermophilic counterparts. These are typical characteristics of a psychrophilic enzyme that enables the adaptation of the SpG6PD1 to the icy environment [34,35].

In *Leuconostoc mesenteroides*, Cosgrove et al. have proposed the catalytic mechanism of LmG6PD. They report that His240 acts as a general base that abstracts the proton from the C_1_-OH of G6P, and the Asp177 was hydrogen bonded to the His240 to stabilize the positive charge forming in the transition state, while His178 interacts with the phosphate moiety of G6P to hold G6P during the reaction [19]. The psychrophilic SpG6PD1 shows similarity with other G6PDs in the conserved catalytic residues (Figure 1), suggesting that the catalytic mechanism of SpG6PD1 is similar to LmG6PD, and the adaptation at low temperatures of SpG6PD1 is achieved through the conformational alterations in those parts of the protein not directly involved in the catalytic residues [36]. 

The kinetic data reveal a significant difference in the *K*_m_ values for G6P in NADP^+^-linked and NAD^+^-linked reactions; the (*K*_mG6P_^NAD+^)/(*K*_m_G6P^NADP+^) ratio of SpG6PD1 was 4.7, which is higher than that obtained for *Streptomyces aureofaciens* [37], *Leuconostoc mesenteroides* [16], and *Methylomonas* M15 [38], but lower than *Thermoanaerobacter tengcongensis* [6]. These strains are classified as dual cofactor-specific or NADP^+^-preferring G6PD enzymes [23]. Thus, SpG6PD1 also belongs to the enzyme category, which utilizes both NADP^+^ and NAD^+^ as cofactors. Moreover, the *k*_cat_/*K*_m_G6P for NADP^+^-linked reactions was found to be considerably higher than that for NAD^+^-linked reactions (31-fold) (Table 3), indicating that SpG6PD1 is an NADP^+^-preferring enzyme. The kinetic data further reveal that the affinity of SpG6PD1 with G6P in NAD^+^-linked reactions is substantially influenced by temperature variations; the *K*_m_G6P values were 675 µM and 4308 µM at 20 °C and 30 °C, respectively. By contrast, slight variations were seen in the values obtained in the NADP^+^-linked reactions (130 µM at 20 °C and 145 µM at 30 °C) (Table 3). This result suggests that the binding of the NADP^+^ and NAD^+^ cofactors to the SpG6PD1 enzyme might change the conformation in different ways. Furthermore, NAD^+^ binding to SpG6PD1 causes a larger conformational change than the binding of NADP^+^, and NADP^+^ stabilizes the structure and provides better thermal stability relative to NAD^+^ [39].

The role of G6PD in protecting cells from oxidants has also been reported. Notably, the activity of G6PDs is remarkably enhanced under conditions containing oxidants such as paraquat, lipid hydroperoxide, and tellurite, which result in an increase of NADPH concentration to combat the oxidative stress [4,40,41]. In *Sphingomonas* sp. PAMC 26621, the NADPH demand is also expected from the presence of antioxidant enzymes in the genome; glutathione reductase and four isoforms of NADPH-dependent thioredoxin reductase indicate that the anti-oxidative potential is truly essential for the survival of this bacterium in cold-induced oxidative stress conditions. Thus, G6PD is expected to play a key role in *Sphingomonas* sp. PAMC 26621 survival under extremely cold habitats such as the Arctic, by generating NADPH.

Most psychrophilic enzymes evolved to obtain the high activity at low temperatures by having a flexible structure surrounding the active site [34,36,42,43]. A similar mechanism is also observed in SpG6PD1, wherein the *K*_m_G6P values are markedly higher, whereas the thermal stability and activation enthalpy (Δ*H*^‡^) are lower than those of mesophilic and thermophilic counterparts (Table 4 and Table 5). Moreover, compared to the cold-adapted and mesophilic counterparts that maintain their initial enzyme activity at moderate temperatures, and thermophilic G6PDs, which retain their activity for 2 h at 70 °C [6,7,10,24,26], the activity of SpG6PD1 was found to be dramatically decreased at moderate temperature (Figure 4). However, SpG6PD1 still retained high enzyme activity at 4 °C (Figure 3). These results suggest that SpG6PD1 possesses a flexible structure and concurrently reduces its stability for activity at low temperature, which is a commonly accepted strategy of psychrophilic enzymes for cold adaptation [35,42,44]. Taken together, the results of the present study indicate that SpG6PD1, an NADP^+^-preferring enzyme, has evolved structural properties that confer a high level of flexibility to adapt to low temperatures, playing a critical role in the survival of *Sphingomonas* sp. PAMC 26621.

## 4. Materials and Methods

### 4.1. Materials

*Sphingomonas* sp. PAMC 26621 was provided by the Polar and Alpine Microbial Collection (PAMC) of the Korea Polar Research Institute (Incheon, Korea) [14]. The pET28b (+) expression vector was purchased from Novagen (Madison, WI, USA), the TA vector was from Enzynomics (Daejeon, South Korea), and the HisTrap, Q Sepharose, and HiTrap desalting columns were from GE Healthcare (Piscataway, NJ, USA). All of the other reagents were purchased from Sigma unless stated otherwise.

### 4.2. Gene Cloning of SpG6PD1

A homology search was carried out using the BLAST program [46]. Sequence alignments were performed using Clustal Omega [47]. The structural model of SpG6PD1 was generated using the Swiss-Model server based on the crystal structure of G6PD from *L. mesenteroides* (PDB ID:1H9A, DOI: 10.1107/S0907444901003420) [48].

The *zwf* gene (Accession number: NZ_AIDW01000018) encoding SpG6PD1 was amplified from the genome of *Sphingomonas* sp. PAMC 26621 using a polymerase chain reaction (PCR) with a forward primer (5′-GTCGCATGCCAATCCGCAC-3′) and a reverse primer (5′-GAAATCAATCGTCCTGCCAGG-3′). The amplified product was subcloned in a TA vector and transformed into *E. coli* DH5α. Next, the *zwf* gene in the TA vector was amplified by PCR using a forward primer: 5′-GTCGCC**ATGG****GGCCAATC**-3′ (*Nco* I site underlined and the N-terminal part of SpG6PD1 in boldface type) and a reverse primer: 5′- GAAATCAAGCTT**GTCCTGCCAG**-3′ (*Hind* III site underlined and the C-terminal part of SpG6PD1 in boldface type). The amplified product was digested with *Hind* III and *Nco* I, and subsequently subcloned in a pET28b (+) vector followed by transformation into *Escherichia coli* BL21 (DE3). The construct was confirmed by DNA sequencing.

### 4.3. Expression and Purification of SpG6PD1

The *E. coli* BL21 (DE3) cells harboring pET28b-*zwf* were cultured at 37 °C in Luria-Bertani (LB) broth containing kanamycin (50 µg/mL). Following isopropyl β-d-1-thiogalactopyranoside induction at cell density OD = 0.6–0.8, the cells were cultured for an additional 10 h at 30 °C and subsequently harvested by centrifugation, washed, and resuspended in buffer A (50 mM of Tris⋅HCl, pH 8.0, 25 mM of NaCl, 0.1 mM of ethylenediaminetetraacetic acid (EDTA), and 5% (*w*/*v*) glycerol). Then, the harvested cells were disrupted by sonication in an ice-water bath, followed by centrifugation of the cell extract at 13000 × g for 30 min at 4 °C. The resultant supernatant was loaded on a 1-mL HisTrap column (GE Healthcare), washed with 10 mL of buffer A, and eluted by using a linear gradient of 20–500 mM imidazole in buffer B (50 mM of Tris⋅HCl, pH 8.0, 500 mM of NaCl, 20 mM of imidazole, and 5% (*w*/*v*) glycerol). The fractions containing G6PD activity were pooled and desalted in buffer A using a 5-mL HiTrap desalting column (GE Healthcare). Next, the recombinant SpG6PD1 protein was purified by anion-exchange column chromatography with a 1-mL Q Sepharose column (GE Healthcare) using a linear gradient of 25–1000 mM KCl in buffer A. All of the purification steps were carried out at 4 °C. The purified enzyme was frozen in liquid N_2_ and stored at –80 °C until further experiments.

### 4.4. Enzyme Assay

The G6PD activity was determined by measuring the production of NAD(P)H at 340 nm. The assay mixture (total volume, 1 mL) consisted of 100 µM of NAD(P)^+^, 2 mM of glucose 6-phosphate, and an appropriate amount of SpG6PD1 enzyme in buffer A. The mixture was incubated at 30 °C for 2 min, and then measured *A_340_* in a 1-cm path length cuvette on a Shimadzu UV-1800 spectrophotometer. One enzyme unit is defined as the amount of enzyme catalyzing the formation of 1.0 µmol of NAD(P)H per min at 30 °C. 

Protein concentration was determined by the Bradford method [49], with bovine serum albumin as standard.

### 4.5. Biochemical Characterization

The optimum pH of SpG6PD1 was determined by performing the enzyme assay in the buffer systems of citric acid–Na_2_HPO_4_ (pH 5.0–5.5), sodium phosphate buffer (pH 6.0–7.0), Tris⋅HCl (pH 7.5–8.5), and Glycine–NaOH (pH 9.0–10.0) for both NADP^+^-linked and NAD^+^-linked reactions. The apparent optimum temperature was determined by measuring the enzyme activity at 4−60 °C in the reaction mixture for 2 min. After measuring the optimum pH and temperature of SpG6PD1 with either NADP^+^ or NAD^+^ as a cofactor, NADP^+^ was employed for characterizing SpG6PD1 throughout the experiments due to the low activity with NAD^+^. To elucidate the effects of different metal ions on the activity of SpG6PD1, 1 mM or 5 mM (final concentrations) of EDTA, Mn^2+^, Zn^2+^, Ni^2+,^ Mg^2+^, Cu^2+^, Ca^2+^, and Fe^3+^ was added into the reaction mixture separately, and SpG6PD1 activity was measured at 30 °C with NADP^+^ as a cofactor. Also, the thermal stability of SpG6PD1 was determined by measuring the residual activity in the reaction mixture containing NADP^+^ at 30 °C for 2 min at various temperatures (4, 20, 30, 40, 50, and 60 °C). Residual enzyme activity was measured every 20 min up to 2 h. The catalytic activity at 30 °C before incubation was considered to be 100%.

### 4.6. Enzyme Kinetics and Thermodynamic Analysis

The Michaelis−Menten constant (*K*_m_) and catalytic rate constant (*k*_cat_) were determined by measuring the activity of SpG6PD1 at varying concentrations of G6P (500–2000 µM) at 20 °C and 30 °C for 2 min. The *V*_max_ and *K*_m_ parameters were calculated by applying the Lineweaver–Burk plot. The activation energy (*E*_a_) of the reaction was determined from the slope of the Arrhenius plot (ln*k*_cat_ versus 1/*T)*. Thermodynamic parameters (Gibbs free energy of activation (Δ*G*^‡^), enthalpy (Δ*H*^‡^), and entropy (Δ*S*^‡^)) were calculated using the following equations, as described in a previous study [35]:

(1) $$\Delta G^{‡}=RT\left[\left(\ln\frac{k_{B}T}{h}\right)-\ln k\right]$$

Δ*H^‡^* = *E*_a_ – *RT* (2)

Δ*S^‡^* = (Δ*H*^‡^ – Δ*G*^‡^)/*T* (3)

where *R* is the gas constant (8.314 J·mol^−1^K^−1^), *k_B_* is the Boltzmann constant (1.3805 × 10^−23^ J·K^−1^), *h* is the Plank constant (6.6256 × 10^−34^ Js), and *k* is the catalytic rate constant.

### 4.7. Spectroscopy Analysis

A 200-µL aliquot of sample (0.3 mg/mL) in buffer A was incubated at the indicated temperature (4, 20, 30, 40, 50, or 60 °C) for 1 h. The temperature-induced unfolding of SpG6PD1 was investigated at the Korea Basic Science Institute (Ochang, Korea) by circular dichroism (CD) spectra using a JASCO J-1500 spectropolarimeter. The spectra were the averages of two scans and were plotted as residual ellipticity (mdeg) against wavelength (nm) using the GraphPad Prism 5 software. 

The fluorescence emission spectra of SpG6PD1 (1 mM) were measured at 25 °C upon incubation at various temperatures for 1 h, with excitation at 280 nm and monitoring the emission spectra between 300–400 nm by using a SCINCO FS-2 fluorescence spectrometer.

## Figures and Tables

**Table 1:** Purification summary table.

Step	Total Protein (mg)	Total Activity (units)	Specific Activity (units/mg)	Yield (%)	Purification (fold)
Cell extract	36.4	11284	310	100	1.0
HisTrap	4.2	7282.9	1717	65	5.5
Q-Sepharose	2.4	6382.8	2700	57	8.7

**Table 2:** Effect of metal ions on SpG6PD1 activity. EDTA: ethylenediaminetetraacetic acid.

Metal Ion	Relative Activity (%)	
	1 mM	5 mM
None	100	100
EDTA	100.8	100.8
Ca^2+^	101.2	104.5
Mg^2+^	107.3	108.0
Ni^2+^	28.6	ND
Zn^2+^	33.0	7.0
Fe^3+^	52.6	4.8
Mn^2+^	99.0	98.8
Cu^2+^	45.8	35.2

Each value represents the mean of triplicate measurements and varies from the mean by not more than 3%. N.D.: not determined.

**Table 3:** Kinetic and thermodynamic parameters of SpG6PD1.

NADP^+^-Linked Reaction						
Temp. (°C)	*K*_m_ (µM)	*k*_cat_ (min^−1^)	*k*_cat_/*K*_m_ (min^−1^·µM^−1^)	Δ*G*^‡^ (kJ·mol^−1^)	Δ*H*^‡^ (kJ·mol^−1^)	*T*Δ*S*^‡^ (kJ·mol^−1^)
20	130 ± 1.5	7018 ± 72	54.0	60.1	27.8	−32.3
30	145 ± 2	8081 ± 48	55.7	61.9	27.8	−34.1
**NAD^+^-Linked Reaction**						
**Temp.** **(°C)**	***K*_m_** **(µM)**	***k*_cat_** **(min^−1^)**	***k*_cat_/*K*_m_** **(min^-1^·µM^−1^)**	**Δ*G*^‡^** **(kJ·mol^−1^)**	**Δ*H*^‡^** **(kJ·mol^−1^)**	***T*Δ*S*^‡^** **(kJ·mol^−1^)**
20	675 ± 9	1196 ± 18	1.80	64.4	54.1	−10.3
30	4308 ± 155	2782 ± 288	0.65	64.6	54.0	−10.6

All of the assays were carried out three times in triplicate.

**Table 4:** Comparison of the thermodynamic parameters of G6PD enzymes.

Source		Temp. (°C)	Δ*G*^‡^ (kJ·mol^−1^)	Δ*H*^‡^ (kJ·mol^−1^)	*T*Δ*S*^‡^ (kJ·mol^−1^)	Reference
*Sphingomonas* sp.	Psychrophile	10	59.5	27.9	−31.6	This study
*Dissostichus mawsoni*	Cold-adapted	0	56.1	36.8	−19.4	[10]
*Chionodraco hamatus*	Cold-adapted	0	56.5	40.6	-15.9	[10]
Human	Mesophile	0	58.2	51.9	-6.3	[10]

**Table 5:** The *K*_m_ value for G6P of G6PD enzymes.

Organism	Cofactor	*K_m_G6P* (µM)	Reference
*Sphingomonas* sp. PAMC 26621	NADP^+^	145	This study
	NAD^+^	674.9	
Human	NADP^+^	47.8	[45]
*Leuconostoc mesenteroides*	NADP^+^	114	[18]
*Ctenopharyngodon idella*	NADP^+^	26	[29]
*Camelus dromedarius*	NADP^+^	66.7	[24]
*Aquifex aeolicus* VF5	NADP^+^	63	[7]

## Supplementary Materials

Supplementary materials can be found at https://www.mdpi.com/1422-0067/20/6/1362/s1.
