# Supplementary material for: Cloning, Expression, and Characterization of a Psychrophilic Glucose 6-Phosphate Dehydrogenase from Sphingomonas sp. PAMC 26621
Source: Int J Mol Sci. 2019 Mar 18;20(6):1362. doi: 10.3390/ijms20061362 (PMC6471386; doi:10.3390/ijms20061362)
Supplement: Supplementary file 1 [file ijms-20-01362-s001.pdf]

Supplementary Table S1: Similarity of SpG6PD1 with other G6PDs.

Organism	Identity (%)	Reference
Sphingomonas elodea	88	[1]
Pseudomonas fluorescens	49	[2]
Escherichia coli	46	[3]
Thermotoga maritima	40	[4]
Thermoanaerobacter tengcongensis	40	[5]
Leuconostoc mesenteroides	35	[6]
Human	32	[7]
Camelus dromedaries	32	[8]
Hordeum vulgare	31	[9]
Aquifex aeolicus VF5	30	[10]

References

1. Vartak, N.B.; Lin, C.C.; Cleary, J.M.; Fagan, M.J.; Saier Jr, M.H. Glucose metabolism in '*sphingomonas elodea*': Pathway engineering via construction of a glucose-6-phosphate dehydrogenase insertion mutant. *Microbiology* **1995**, *141*, 2339-2350.

2. Maleki, S.; Mærk, M.; Valla, S.; Ertesvåg, H. Mutational analyses of glucose dehydrogenase and glucose-6-phosphate dehydrogenase genes in *pseudomonas fluorescens* reveal their effects on growth and alginate production. *Appl. Environ. Microbiol.* **2015**, AEM. 03653-03614.

3. Fuentealba, M.; Muñoz, R.; Maturana, P.; Krapp, A.; Cabrera, R. Determinants of cofactor specificity for the glucose-6-phosphate dehydrogenase from *escherichia coli*: Simulation, kinetics and evolutionary studies. *PloS one* **2016**, *11*, e0152403.

4. Hansen, T.; Schlichting, B.; Schönheit, P. Glucose-6-phosphate dehydrogenase from the hyperthermophilic bacterium *thermotoga maritima*: Expression of the g6pd gene and characterization of an extremely thermophilic enzyme. *FEMS Microbiol Lett* **2002**, *216*, 249-253.

5. Li, Z.; Jiang, N.; Yang, K.; Zheng, J. Cloning, expression, and characterization of a thermostable glucose-6-phosphate dehydrogenase from *thermoanaerobacter tengcongensis*. *Extremophiles* **2016**, *20*, 149-156.

6. Lee, W.T.; Flynn, T.; Lyons, C.; Levy, H. Cloning of the gene and amino acid sequence for glucose 6-phosphate dehydrogenase from *leuconostoc mesenteroides*. *J. Biol. Chem.* **1991**, *266*, 13028-13034.

7. Au, S.W.; Gover, S.; Lam, V.M.; Adams, M.J. Human glucose-6-phosphate dehydrogenase: The crystal structure reveals a structural nadp+ molecule and provides insights into enzyme deficiency. *Structure* **2000**, *8*, 293-303.

8. Saeed, H.; Ismaeil, M.; Embaby, A.; Ataya, F.; Malik, A.; Shalaby, M.; El-Banna, S.; Ali, A.A.M.; Bassiouny, K. Overexpression, purification and enzymatic characterization of a recombinant arabian camel *camelus dromedarius* glucose-6-phosphate dehydrogenase. *Protein Expr. Purif.* **2015**.

9. Cardi, M.; Chibani, K.; Castiglia, D.; Cafasso, D.; Pizzo, E.; Rouhier, N.; Jacquot, J.-P.; Esposito, S. Overexpression, purification and enzymatic characterization of a recombinant plastidial glucose-6-phosphate dehydrogenase from barley (*hordeum vulgare* cv. Nure) roots. *Plant Physiol. Biochem.* **2013**, *73*, 266-273.

10. Iyer, R.B.; Wang, J.; Bachas, L.G. Cloning, expression, and characterization of the gsda gene encoding thermophilic glucose-6-phosphate dehydrogenase from *aquifex aeolicus*. *Extremophiles* **2002**, *6*, 283-289.
